# Clinical outcomes of prolonged infusion (extended infusion or continuous infusion) versus intermittent bolus of meropenem in severe infection: A meta-analysis

**DOI:** 10.1371/journal.pone.0201667

**Published:** 2018-07-30

**Authors:** Zhenwei Yu, Xiaoping Pang, Xuqi Wu, Chunlei Shan, Saiping Jiang

**Affiliations:** 1 Sir Run Run Shaw Hospital, College of Medicine, Zhejiang University, Hangzhou, Zhejiang, China; 2 Taizhou Enze Medical Center (Group) Enze Hospital, Taizhou, Zhejiang, China; 3 Huzhou Third Municipal Hospital, Huzhou, Zhejiang, China; 4 The First Affiliated Hospital, Zhejiang University, Hangzhou, Zhejiang, China; Toranomon Hospital, JAPAN

## Abstract

**Background:**

Meropenem exhibits time-dependent antimicrobial activity and prolonged infusion (PI) (extended infusion or continuous infusion, EI or CI) of meropenem can better achieve pharmacodynamics target when comparing with intermittent bolus (IB). However, the clinical outcomes between two groups remain inconclusive.

**Objective:**

To evaluate current published literatures by meta-analysis to ascertain whether PI of meropenem can improve clinical outcomes.

**Methods:**

Medline, Cochrane database and EMBASE were searched. Randomized control trails (RCT) and observational studies which compared the clinical outcomes of PI and IB groups were included and evaluated for quality. The data of studies were extracted and meta-analysis was performed using Revman 5.3 software.

**Results:**

Six RCTs and 4 observation studies with relatively high quality were included in this analysis. Compared to IB group, PI group had a higher clinical success rate (odd ratio 2.10, 95% confidence interval 1.31–3.38) and a lower mortality (risk ratio 0.66, 95% confidence interval 0.50–0.88). The sensitivity analysis showed the results were stable.

**Conclusion:**

PI of meropenem was associated with a higher clinical improvement rate and a lower mortality. It is recommended for patients with severe infection or infected by less sensitive microbial.

## Introduction

Meropenem has strong antimicrobial activity and wide antibiotic spectrum, and is always used to treat for serious infections in clinical therapy [1]. In common with other beta lactams, meropenem exhibits primary time-dependent antimicrobial activity, and the pharmacokinetics/pharmacodynamics (PK/PD) index, which best predicts clinical efficiency is the duration of maintenance of the drug concentration above minimum inhibitory concentration (MIC) for the pathogen (referred as %T>MIC) during each dosing interval [2].

Optimal patient outcome of infection treatment was most likely to occur when pharmacokinetic/pharmacodynamics (PK/PD) targets are achieved, which are associated with maximal antibiotic activity [3]. The T>MIC requiring for carbapenem has been reported to be at least 40% [4], and a T>MIC of 100% displayed significantly greater clinical and bacterial outcomes in patients with severe infection[5]. Prolonged infusion (PI), including extended infusion (EI) and continuous infusion (CI) of beta lactams, can prolong the T>MIC and improve antibacterial activity [6,7].

Although most of the studies reported that PI of meropenem can better achieve at the PK/PD target comparing with intermittent bolus (IB), especially in patients infected with less sensitive pathogen [8–10]. However, the clinical outcomes between two groups remain inconclusive [11]. Thus, the aim of this study was to evaluate current published literatures by meta-analysis to ascertain whether if PI of meropenem can improve clinical outcomes.

## Methods

This systematic review and meta-analysis followed the PRISMA (Preferred Reporting Items for Systematic Reviews and Meta-Analyses) protocol[12].

### Literature search strategy

We have searched Medline, Cochrane database and EMBASE database till Oct 18, 2017. The searching item was followed as: (Meropenem OR beta-lactam OR carbapenem) AND (extended OR continuous OR prolonged OR intermittent OR interval OR discontinuous OR bolus OR pulse) AND (infusion OR administration OR dosing). No language restriction was applied in the study.

### Study selection

Two reviewers (XPP and XQW) searched the literatures and selected the studies independently. The discrepancies between reviewers were resolved by discussions with a third reviewer (ZWY). Both randomized controlled clinical trials (RCTs) and observational studies met the following criteria were included for meta-analysis: (i) severe infections treated by meropenem, (ii) compared the clinical outcomes of PI with IBs’. Studies which only reported pharmacokinetic results or inextractable data were excluded. Duplicated publications were excluded.

### Data extraction

Two reviewers extracted (XPP and XQW) the data independently. The discrepancies between reviewers were resolved by discussions with a third reviewer (ZWY). The following details were extracted: study design, patients’ characteristics, dosing regimen, clinical outcomes (clinical improvement, mortality, microbiological eradication and so on).

### Quality assessment

The qualities of included studies were assessed by two reviewers independently (XPP and XQW). The conflict results were judged by a third reviewer (ZWY). The quality of RCTs and observation studies was assessed by modified Jadad score and Newcastle-Ottawa system (NOS)[13], respectively.

### Statistical analysis

The meta-analysis was carried on Review Manager 5.3 software (Copenhagen: The Nordic Cochrane Centre, The Cochrane Collaboration, 2014.). Risk ratio (RR) and 95% confidential interval (CI) were calculated for mortality, and odds ratio (OR) and 95% CI were calculated for other outcomes. Heterogeneity among studies was investigated using I^2^ test (I^2^ >50% was defined to indicate significant heterogeneity). Mantel-Haenszel fixed effect model was used when no significant heterogeneity existed among studies; otherwise, a random model was used. Sensitivity analyses were performed by exclusion of each study one by one to evaluate the stability of results without estimation of bias from individual study. This process allowed for identification of any single article which may have a great influence on the final result. Publication bias was evaluated by funnel plots when included studies were more than 10.

## Results

### Literature search and study description

**Fig 1:**
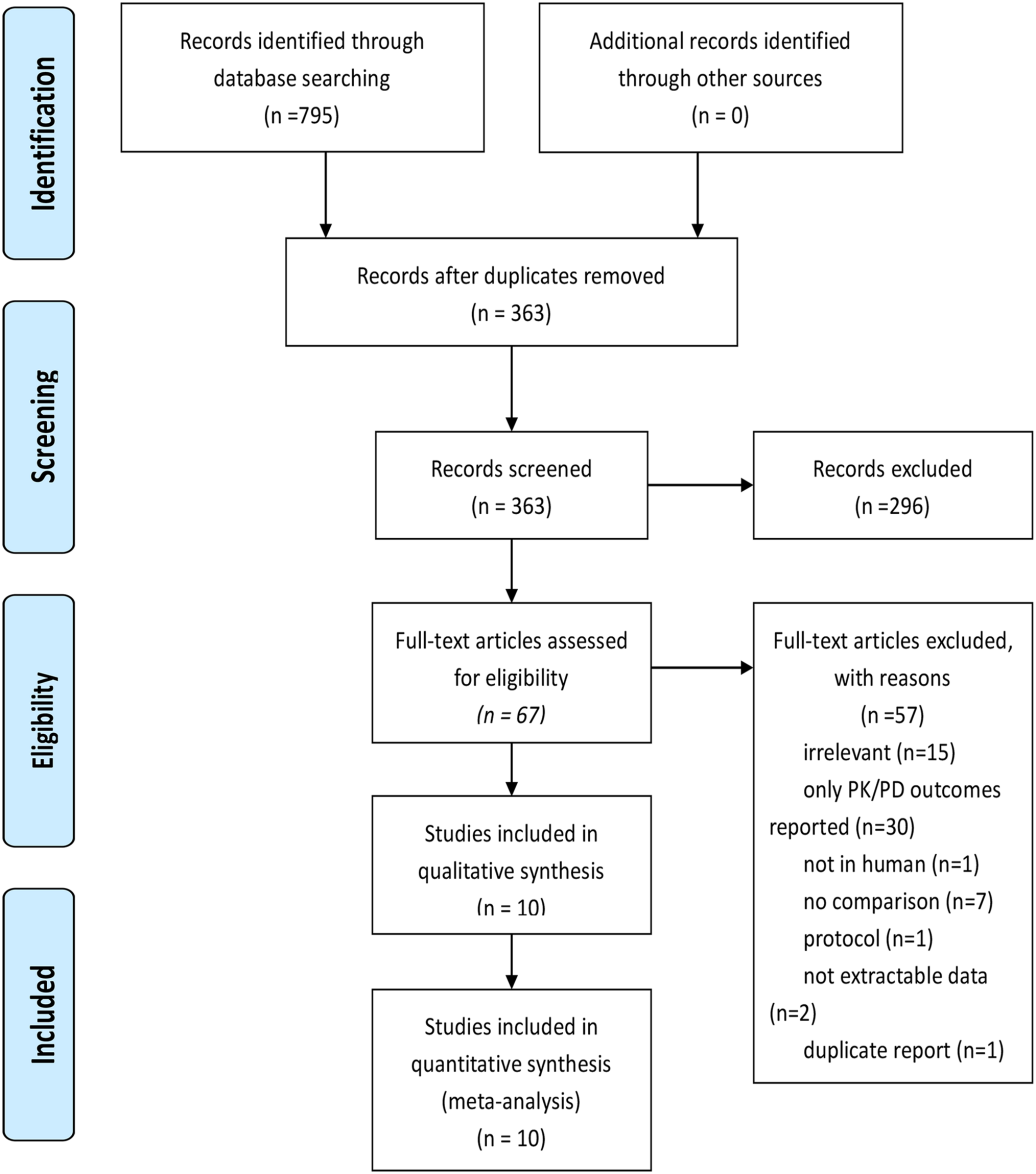
Flow diagram of selection process of the included studies.

The literature search process was shown in Fig 1. The search strategy yielded 363 titles and abstracts, and a total of 10 studies, including 6 RCTs[14–19] and 4 observation studies[20–23]. All the included studies were published in English except one in Chinese [18]. The details were shown in Table 1. Two of the studies compared PI with IB of beta lactam including meropenem but the clinical outcomes of meropenem was not reported in an extractable form [16,20]. The required data of these two studies were kindly provided by the authors. All the studies included ICU patients except one included hematology department patients who presented fever after receiving hematopoietic stem-cell transplantation or induction chemotherapy for acute myeloid leukemia. The infected sites were various and the primary infected pathogens were gram negative. Each study evaluated the severity of underling illness of the patients using APACH II or SOFA scores and there was no significant difference between two infusion groups. The sample sizes of included studies ranged from 30 to 214 and a total of 951 patients were included into the analysis.

**Table 1:** Main characteristics of included studies.

Study	Study design	Patient population	Infection type	Organism isolated	Sensitivity analysis	Sample size (T/C)	Gender ^a^	Age (T/C)	Dosage regimens	Jadad or NOS score
Abdul-Aziz a 2016 [20]	Observational, retrospective	68 ICUs across 10 countries	Various	Various	Not mentioned.	18/41	44/23; 69/46 ^b^	56 (47–75)/ 64 (48–74)	NR	8
Abdul-Aziz b 2016 [14]	RCT	ICU, Malaysian	Severe sepsis	Various	Not mentioned.	21/21	46/24; 50/20	54 (42–63)/ 56 (41–68)	CI: LD 1g over 30 min, then 1g over 480 min; IB: 1 g over 30 minutes, q8h	5
Chytra 2012[15]	RCT	ICU, Czech	Various	Various	MIC determined	120/120	78/42; 83/37	44.9 ± 17.8/ 47.2 ± 16.3	PI: 2g LD; 4 g over 24 h IB: 2 g q8h	2
Dulhunty 2015 [16]	RCT	25 ICUs	Various	Various	Mentioned, not specified	63/60	130/82; 135/85 ^b^	64 (54–72)/ 65 (53–72)	The median 24-hour dose on Day 1 was 3.0 g. PI: continuous infusion IB: over 30 minutes	7
Feher 2014 [21]	Observational, retrospective	Hematology department, Spanish	Fever^c^	NR	Mentioned, not specified	76/88	44/32; 42/46	44.0 (32.5–56.0)/ 49.5 (37.0–57.5)	PI:1 g q8 h over 4 h IB:1 g q8 h over 30 minutes	8
Lorente 2006 [22]	Observational, retrospective	ICU, Spain	VAP	Gram-negative Bacilli	MIC determined	42/47	33/9; 38/9	57.25±19.0/ 56.46±18.8	PI:1 g q6 h over 6 h IB:1 g q6 h over 30 min	7
Shabaan 2017 [17]	RCT	NICU, Egypt	Sepsis	Gram-negative	Mentioned, not specified	51/51	25/25; 30/21	8d (6–13)/ 6d (5–15)	20mg/kg q8h and 40mg/kg q8h in meningitis and pseudomonas infection PI: over 4h, IB: over 30 min	4
Wang 2009 [23]	Observational, retrospective	ICU, China	VAP	MDR A. Baumannii	MIC determined	15/15	10/5; 9/6	44.33 ± 21.0/ 39.67 ± 21.6	PI: 0.5g q6h over 3h IB: 1 g q8h over 1h	7
Wang 2014 [18]	RCT	ICU, China	HAP	Various	Mentioned, not specified	38/40	25/13; 34/6	63.5±15.3/ 57.2±19.5	PI: First dose LD 0.25g over 10 min, 0.75g over 3h; then 1g q8h over 3h IB:1g q8h over 30 min	2
Zhao 2017 [19]	RCT	ICU, China	Various	Various	MIC determined	25/25	10/15; 11/14	68.0 ± 15.4/ 67.0 ± 12.2	PI: LD 0.5 g over 30 min; 3 g over 24 h IB: First dose 1.5 g over 30 min; 1 g q8h over 30 min	4

Abbreviations: T/C: prolonged infusion group versus control group; NR: not reported; RCT: randomized control clinical trial; ICU: intensive care unit; NICU: neonate intensive care unit; LD: loading dose; CI: continuous infusion; PI: prolonged infusion; IB: intermittent bolus; VAP: ventilator associated pneumonia; HAP: hospital acquired pneumonia; MDR: multi-drug resistance; NOS: Newcastle-Ottawa system

^a^ the data of gender was presented as male/female in prolonged infusion group; male/female in control group.

^b^ These two studies had compared prolonged infusion versus intermittent bolus of beta lactam including meropenem, but the clinical outcome of meropenem had not been reported in the article or reported in extractable form. The data was kindly provided by the authors and the details were referred to the whole study population except sample size.

^c^ Neutropenic patients who presented with fever after receiving hematopoietic stem-cell transplantation or induction chemotherapy for acute myeloid leukaemia.

### Quality of included studies

As shown in Table 1, the qualities of 6 studies includeding RCTs were assessed and 4 of them yielded Jadad Scores above 4 and, definding as high quality. The main shortage of study design was blinding, that was only one study designed double blinding trial adequately [16]. Four observational studies were included and two studies were prospective while the other two were retrospective. Eight factors were used to assess the quality according to NOS and yielded high quality. Two studies missed one indicator and the other two were adequate in all criteria. Overall, the qualities of included studies were relatively high.

### Clinical success

**Fig 2:**
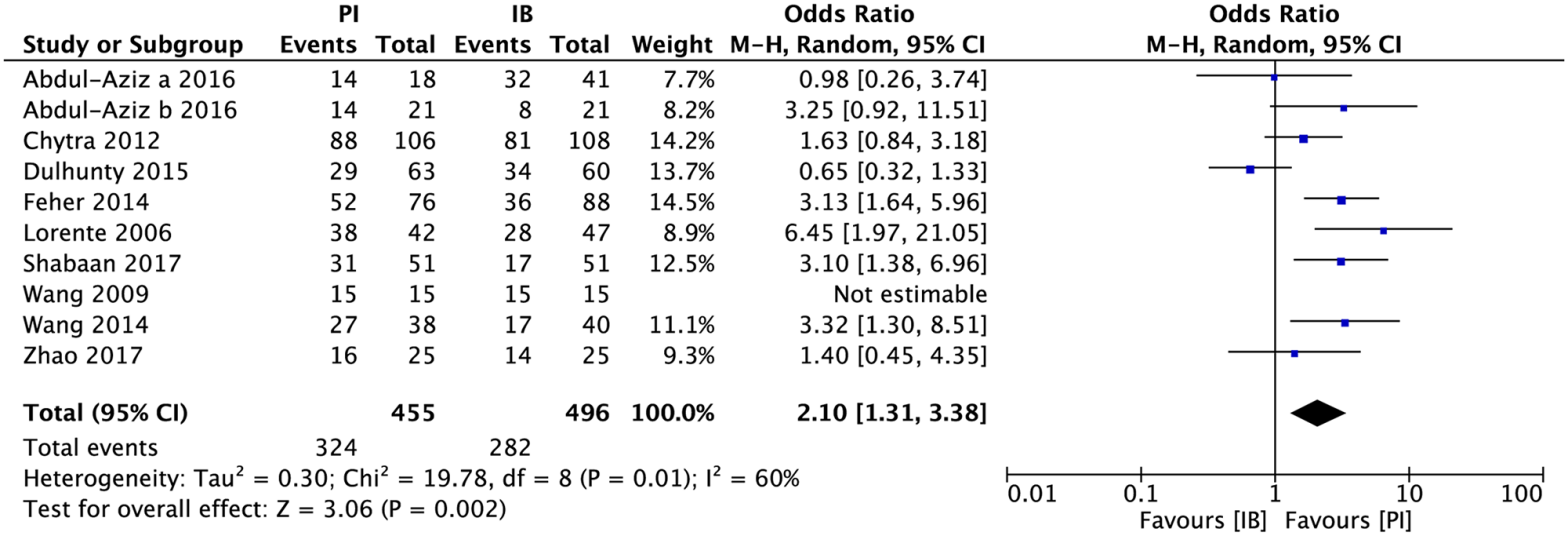
Forest plots depicting the odds ratio of clinical success rate of patients receiving extended or continuous infusion vs intermittent of meropenem.

All the included studies reported clinical success rate, while some studies defined the success as clinical cure (as the disappearance of all signs and symptoms related to the infection) and clinical improvement (as partial resolution of clinical signs and symptoms related to the infection) [15,19,21], but some defined as clinical cure only [14,16,17,20,22]. Significant heterogeneity was found among studies and random model was used. The analysis result showed that extended or continuous infusion had a significantly higher cure rate than intermittent infusion (Fig 2, 951 patients, OR = 2.10, 95%CI 1.31–3.38). The result of stratification analysis was shown in S1, S2 and S3 Figs.

### Mortality

**Fig 3:**
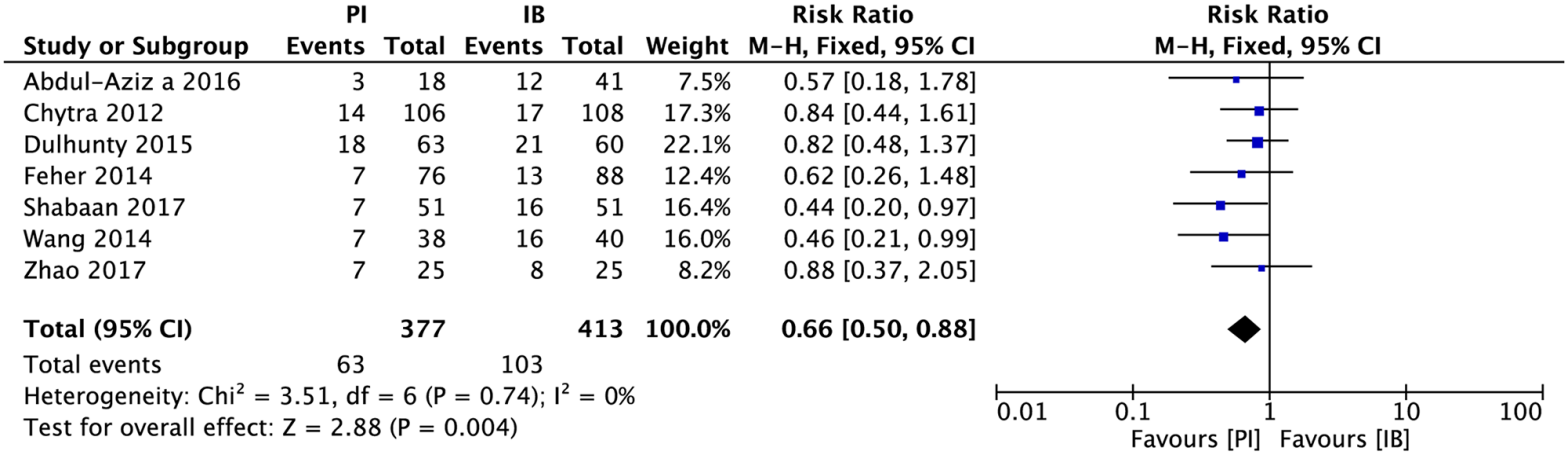
Forest plot depicting the risk ratio of mortality of patients receiving extended or continuous infusion vs intermittent of meropenem.

Seven studies, including 5 RCTs[15–19] and 2 observational studies [20,21] reported mortality at different time points. In the current research, mortalities at the endpoint of each study were extracted for analysis. No heterogeneity was found among studies. The mortality of extended or continuous infusion group was significantly lower than intermittent group (Fig 3, 790 patients, RR = 0.66, 95%CI 0.50–0.88).

### Bacterial eradication

**Fig 4:**
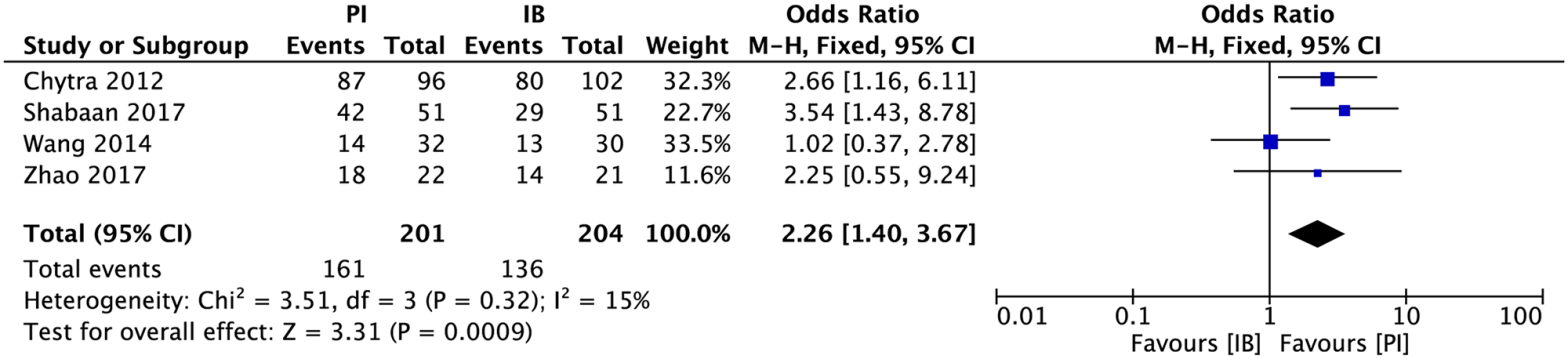
Forest plot depicting the odds ratio of microbial eradication of patients receiving extended or continuous infusion vs intermittent of meropenem.

Four RCTs[15,17–19] had reported bacterial eradication rate and no significant heterogeneity was found among the studies. The results showed that bacterial eradication rate of continuous infusion group was significantly higher than that of intermittent infusion group (Fig 4, 405 patients, OR = 2.26, 95% CI 1.40–3.67).

### Other outcomes

There were 5 studies [15,17–19,21] reporting the lengths of stay (LOS) in hospital/ICU of each group, while 4 of which reporting data in an inextractable form and the meta-analysis can not be run[15,17,19,21]. Four of the studies have not found significant difference between two groups[17–19,21], while Chytra’s study reported that continuous infusion of meropenem can decrease the LOS in ICU[15].

There were 3 studies reporting the adverse events (AE) during the therapy but the results were not consistent[14,17,18]. No AE occurred in Abdul-Aziz’s study[14], but the total AE rates in Wang’s study were around 40% and no significant difference was found between two groups[18]. Shabaan reported that acute kidney injuries were more common in IB group[17].

There were 3 studies[18,19,23] reporting the duration of meropenem treatment and no significant heterogeneity was found among studies. The result of meta-analysis had shown that there was no significant difference between prolonged infusion and intermittent infusion (S4 Fig).

### Sensitivity analysis and publication bias

The sensitivity analyses were carried on by exclusion of individual study one by one. The results of sensitivity analysis of two main outcomes (clinical success and mortality) had shown no substantial difference among the estimates. Publication bias might exist due to an asymmetric funnel plot achieved (S5 Fig).

## Discussion

Studies for PI of beta-lactam including meropenem are numerous. These studies suggest that PI achieves a greater likelihood of achieving PK/PD targets than standard IB in critically ill patients[24,25]. Recent studies have sought to investigate the clinical value of these PI with RCTs, suggesting a potential advantage of PI for patients with severe sepsis[26,27]. The results of our meta-analysis had shown that PI of meropenem was associated with a higher clinical improvement rate and a lower mortality than IB. While some meta-analysis comparing PI and IB of beta lactam have not been able to quantify definitive advantage for either PI or IB, these studies have often not been stratified for patients with altered PK or reduced susceptibility[28–30]. MIC ranges of infected pathogen were very important to the outcomes, patients infected by microorganism with low MIC had obtained target PK/PD thresholds using standard infusion of antibiotics.[31,32]

Both observational and RCTs studies were included in this meta-analysis. Observational studies included yield high quality. Similarly, included RCTs were also relatively high quality but 2 of them[15,18] got modified Jadad scores below 4. It is because that the PI process is difficult to achieve blind to investigators or patients. Only one study designed as double blind trail and used 0.9% sodium chloride as placebo in the IB group[16], but critically ill patients always needed to be restricted with fluid[33]. Other RCTs were designed as open trail or single blind trail. Over all, the quality of included studies was relatively high, which makes the results of this analysis reliable.

The clinical success rate of PI group is significantly higher than that of IB group according to the analysis result, but there is heterogeneity existing among studies. The possible reason may be the definition of clinical success. Some studies defined clinical improvement as clinical success as well as clinical cure, while some studies didn’t. There are still some other confounding factors. The administration methods in PI group were different from each other. The subgroup analysis showed that 3-hour prolonged infusion had higher clinical success rate than 24-hour continuous infusion (S2 Fig). The stability of meropenem is also an important issue. It is reported that degradation of generic meropenem was both time and temperature dependent, and the aqueous solutions were stable for up to 8 hours in the temperature range between 25°C and 35°C, while for up to 5 hours at 40°C[34]. But only one of the 24-hour infusion studies had specified how to account the stability problem[19]. Some studies used different dose between PI and IB group. The subgroup analysis showed that equivalent dose exhibited more differences between PI and IB groups than non-equivalent dose.

The mortalities of each study were much more consist than the result of clinical success rate. The meta-analysis result showed PI groups had lower mortality than IB. It is noticed that result of mortality and clinical success rate in Dulhunty’s study were conflicting. That may due to the definition of clinical success mentioned above and the random effect caused by small sample size.

Sensitivity analysis was used to evaluate the stability of results, especially when heterogeneity existed. The results of sensitivity analysis of clinical success rate and mortality had shown no substantial difference among the estimates, which elevated the reliability of the results.

The bacterial eradication rate was significantly higher in CI group according to the result of meta-analysis. It can be explained that CI of meropenem can result in higher tissue concentration and then exhibiting better microbiological effect [35,36]. The results of LOS and AE among studies were not consistent which needs to be verified in further studies. The analysis results had shown that there was no significant difference on duration of meropenem treatment between two infusion groups. But the meropenem treatment can be ceased under many circumstances and the three studies had not specified the reason. The included population was also relatively small and this result needs to be further investigated.

Furthermore, there are some other limitations. The sample sizes were relatively small in most included studies. Many confounding factors haven’t been controlled or reported, such as type and site of infection, sensitivity of pathogen, co-use of antibiotics, and so on. Moreover, bias may exist because of heterogeneity among included studies and asymmetric funnel plot.

In conclusion to the current evidence, PI of meropenem was associated with a higher clinical improvement rate and lower mortality. It is recommended to patients with severe infection or infected by less sensitive microbial. Furthermore, well-designed RCTs evaluating prolonged infusion and intermittent infusion of meropenem are still needed.

## Supporting information

S1 Fig Subgroup analysis of definition of clinical success of patients receiving prolonged infusion (PI) vs intermittent bolus (IB) of meropenem. ND: not defined.
(TIF)

S2 Fig Subgroup analysis of administration method on clinical success of patients receiving prolonged infusion (PI) vs intermittent bolus (IB) of meropenem. CI: continuous infusion; EI: extended infusion.
(TIF)

S3 Fig Subgroup analysis of dose equivalency on clinical success of patients receiving prolonged infusion (PI) vs intermittent bolus (IB) of meropenem. NR: not reported.
(TIF)

S4 Fig Forest plot depicting the mean difference of duration of meropenem treatment of patients receiving prolonged infusion (PI) vs intermittent bolus (IB) of meropenem. (TIF)

S5 Fig Funnel plot to evaluate publication bias. (PDF)

S1 Appendix PRISMA checklist. (DOC)
