# Supplementary figures and images for: Clinical outcomes of prolonged infusion (extended infusion or continuous infusion) versus intermittent bolus of meropenem in severe infection: A meta-analysis
Source: PLoS One. 2018 Jul 30;13(7):e0201667. doi: 10.1371/journal.pone.0201667 (PMC6066326; doi:10.1371/journal.pone.0201667)

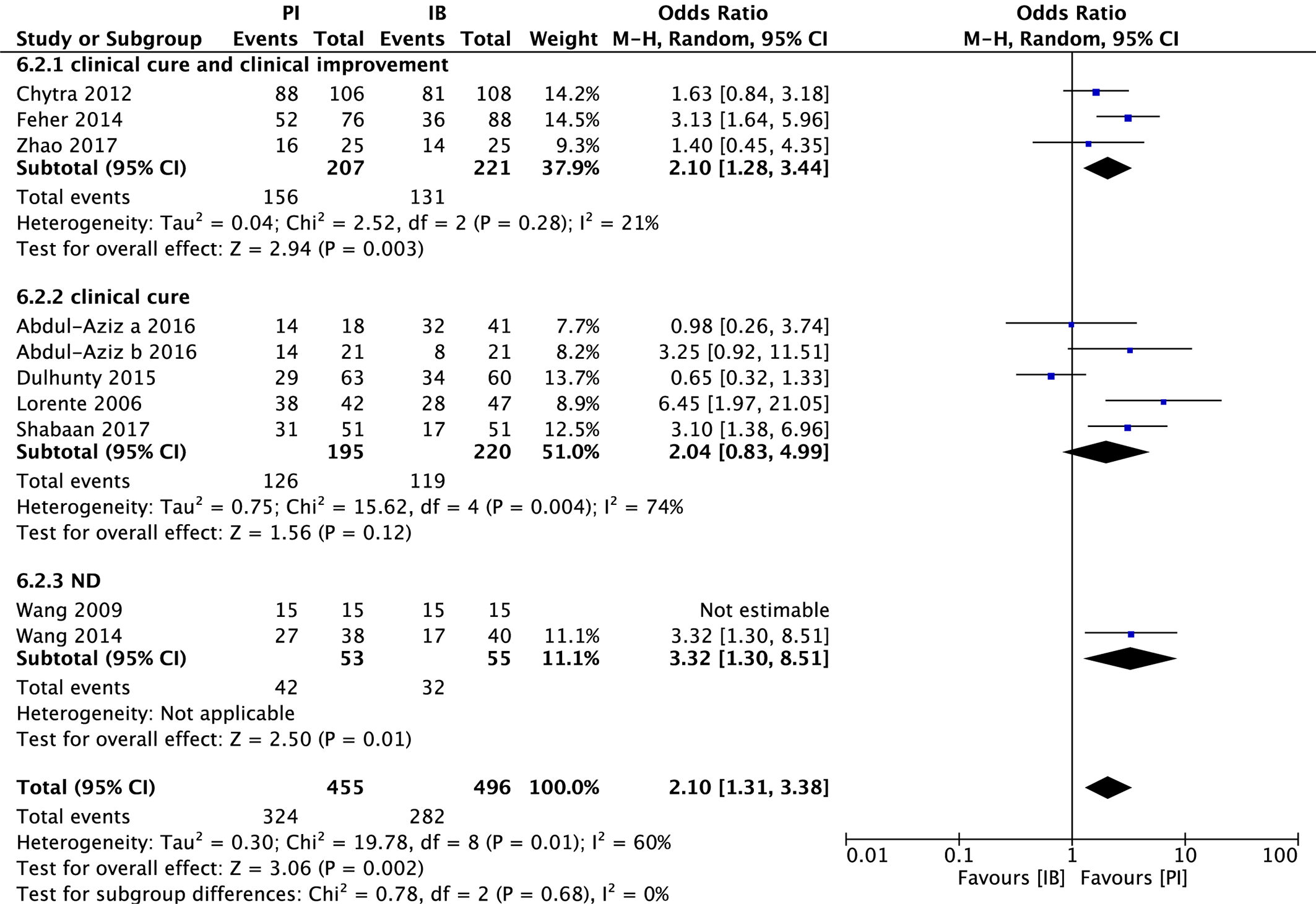

Supplement: S1 Fig — ND: not defined. (TIF) [file pone.0201667.s001.tif]

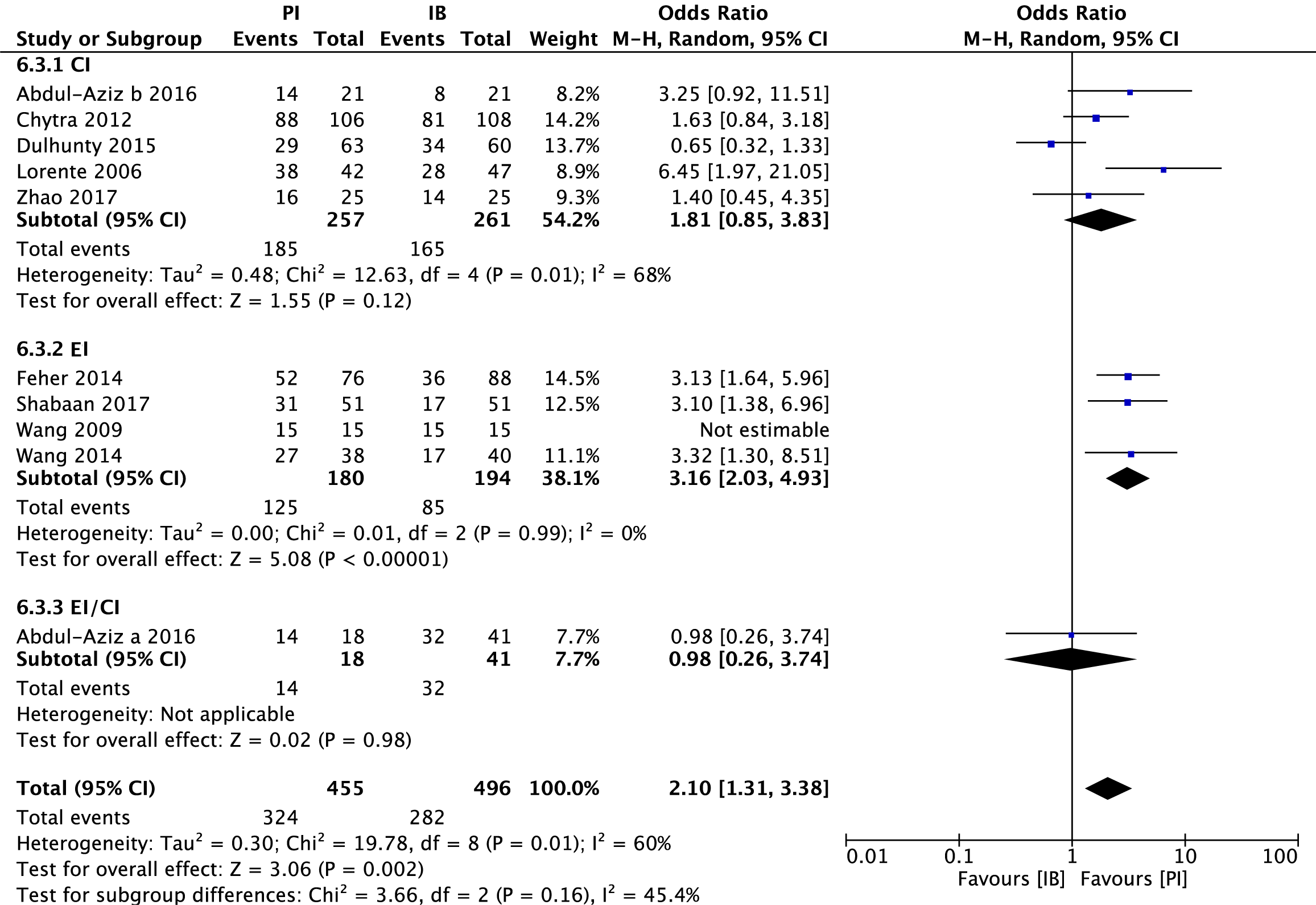

Supplement: S2 Fig — CI: continuous infusion; EI: extended infusion. (TIF) [file pone.0201667.s002.tif]

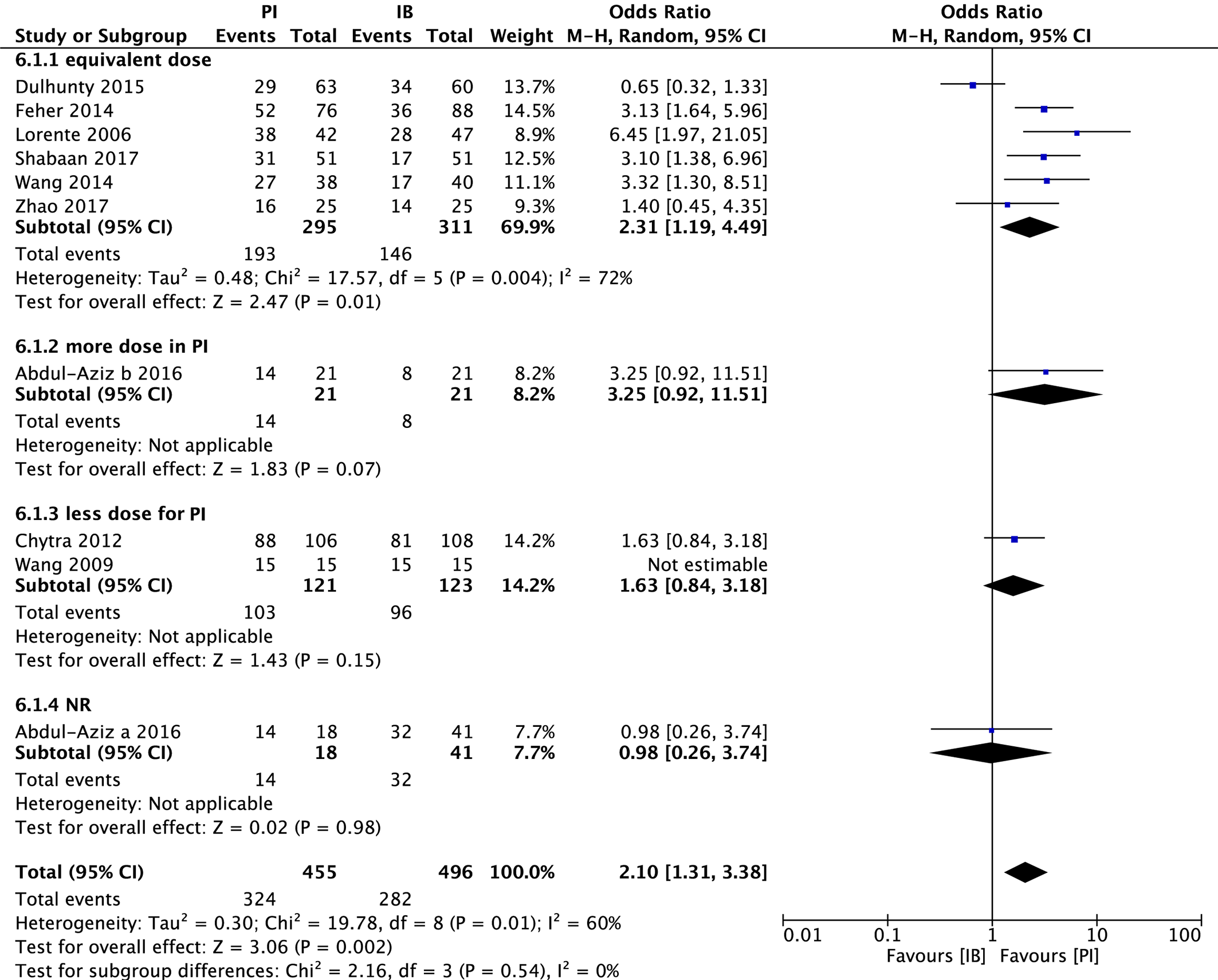

Supplement: S3 Fig — NR: not reported. (TIF) [file pone.0201667.s003.tif]

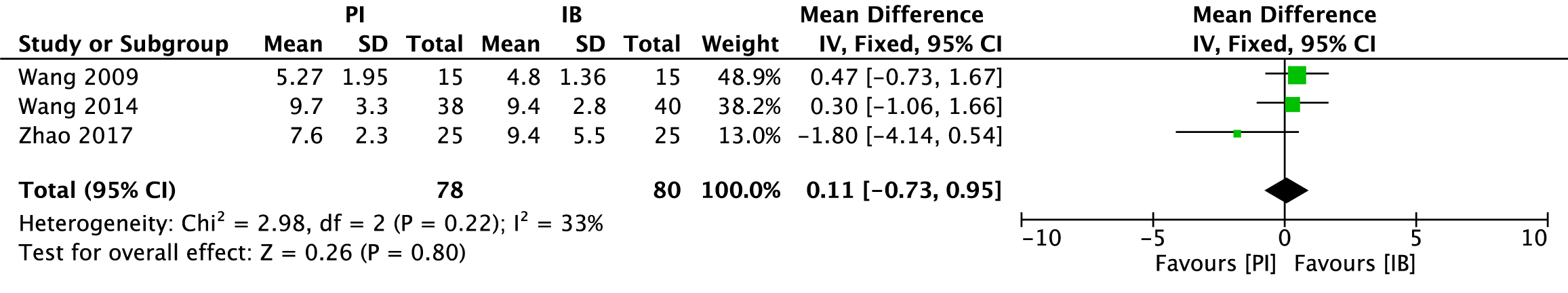

Supplement: S4 Fig — (TIF) [file pone.0201667.s004.tif]

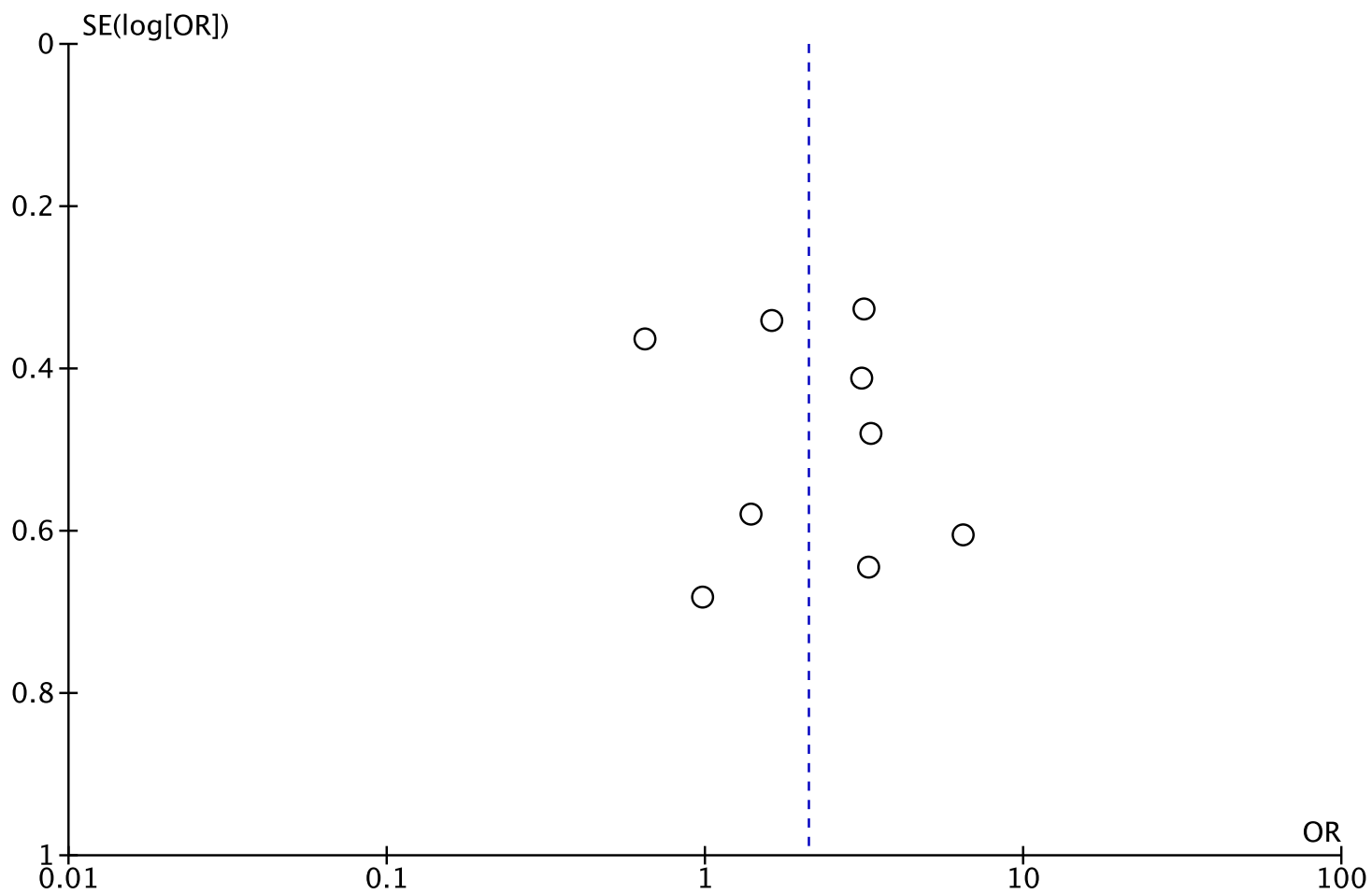

Supplement: S5 Fig — (PDF) [file pone.0201667.s005.pdf]
